# Magnesium, fibrinolysis and clotting interplay among children and adolescents with type 1 diabetes mellitus; potential mediators of diabetic microangiopathy

**DOI:** 10.1038/s41387-025-00368-9

**Published:** 2025-04-01

**Authors:** Dalia N. Toaima, Kholoud S. Abdel-Maksoud, Heba M. Atef, Nouran Y. Salah

**Affiliations:** 1 Department of Pediatrics, Faculty of Medicine, Ain shams University, Cairo, Egypt; 2 Ministry of Health and Population, Cairo, Egypt; 3 Department of Clinical Pathology, Faculty of Medicine, Ain shams University, Cairo, Egypt

**Keywords:** Type 1 diabetes, Diabetes complications

## Abstract

**Background and aim:**

Hypomagnesemia and clotting disorders have been reported among people with diabetes especially those with type 2 diabetes (T2DM). Magnesium plays a crucial role in hemostasis and hypomagnesemia was found to increase the thrombotic risk. The patho-mechanism linking magnesium, clotting disorders, and diabetic microangiopathy in T1DM remains to be unraveled. Hence this study aimed to assess the magnesium level among children and adolescents with T1DM compared to healthy controls and to correlate it with coagulopathy markers and diabetic microangiopathy.

**Methods:**

Forty-six children and adolescents with T1DM & 46 controls were assessed for serum magnesium, prothrombin time (PT), activated-partial thromboplastin time (aPTT), plasminogen activator inhibitor-1 (PAI-1) and HbA1c. The Toronto clinical scoring system, fundus, urinary microalbumin, and serum fasting lipids were used to assess diabetic microangiopathy.

**Results:**

Children and adolescents with T1DM have significantly lower magnesium, PT, aPTT, and significantly higher PAI-1 than controls (*p*<0.001), this is more evident in those having microangiopathy than those without (*p*<0.001). Serum magnesium is positively correlated with PT, aPTT, and HDL and negatively correlated with insulin daily dose, PAI-1, HbA1c, triglycerides, and urinary microalbumin. Multivariate-logistic regression revealed that diabetes duration, HbA1c, PT, aPTT, PAI-1, and urinary microalbumin were independently associated with serum magnesium among children and adolescents with T1DM (p<0.05).

**Conclusion:**

Children and adolescents with T1DM have lower magnesium levels than controls; that is more pronounced among those having microangiopathy. Low serum magnesium is associated with poor glycemic control, coagulopathy, and diabetic microangiopathy among children and adolescents with T1DM. Magnesium supplementation combined with standard insulin therapy in pediatric patients with T1DM is recommended for better glycemic control and prevention of diabetic microangiopathy.

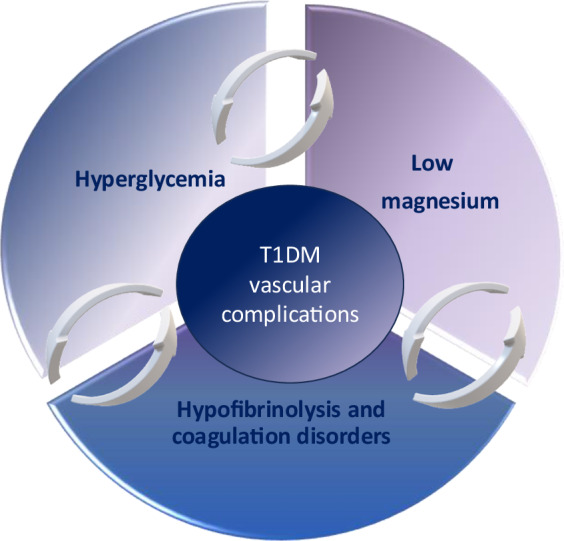

## Introduction

Magnesium is an essential micronutrient that acts as a cofactor for approximately 600 enzymes and an activator for a further approximately 200 enzymes. It plays a vital role in the homeostasis of other minerals, such as sodium, potassium, and calcium, as well as in the formation, transfer, storage, and utilization of adenosine triphosphate (ATP). Moreover, it has many physiological functions, including DNA and RNA stability maintenance, and regulation of cellular proliferation, bone metabolism, neuromuscular functioning, inflammation, and hemostasis [1].

Magnesium is known to strongly influence the coagulation cascade. The addition of magnesium in vitro results in the inability of blood to clot [2]. Although not routinely used, magnesium sulfate has been successfully used during blood collection as an alternative to ethylenediaminetetraacetic acid and citrate [3]. Magnesium is thought to interfere with blood clotting by competing with calcium ions [4].

Magnesium homeostasis is closely linked to blood insulin. On one hand, insulin affects renal tubular magnesium reabsorption through upregulating protein expression and transient receptor potential channel (TRPM) 6 activity in the distal convoluted tubule [5]. On the other hand, magnesium plays a role in insulin release regulation and energy metabolism [6].

Individuals with T1DM were found to have a reduced plasma magnesium concentration compared with controls. Moreover, an inverse relationship was found between serum magnesium and glycemic control [7]. Magnesium deficiency is hypothesized to affect glycemic control by altering cellular glucose transport, reducing pancreatic insulin secretion, and defective post-receptor insulin signaling [8].

Studies have linked reduced serum magnesium to various diabetic complications. Reduced magnesium is thought to influence diabetic vascular complications through altering inositol transport, increasing the levels of tumor necrosis factor-alpha, and increasing the formation of advanced glycosylation end-products [9].

Plasmin; the enzyme responsible for fibrin breakdown; is generated from plasminogen through the action of the tissue plasminogen activator (tPA) [10]. Plasminogen activator inhibitor (PAI-1) is produced by endothelial cells, platelets, and adipose tissue. PAI-1 forms a stable complex with tPA, inhibiting its action thereby inhibiting excessive clot lysis [11].

Elevated PAI-1 level was found to be associated with an increased risk of coronary vascular diseases, obesity, insulin resistance, and T2DM. However, these associations are less evident when addressing T1DM [12].

Given that people with T1DM have an increased thrombotic risk and magnesium is known to play an essential role in coagulation control; this study aims to assess the level of serum magnesium among children and adolescents with T1DM compared to healthy matched controls and its correlation with glycemic control, coagulopathy markers including prothrombin time (PT), activated partial thromboplastin time (aPTT), plasminogen activator inhibitor-1 (PAI-1) and diabetic vascular complications.

## Materials and methods

### Participants

Forty-six children and adolescents with T1DM were recruited from the regular attendees of the Pediatric and Adolescents Diabetes Unit (PADU), Ain Shams University, and 46 healthy children were included as a control group from the healthy siblings of attendees of the outpatient clinic. They were matched for age and gender. Participants were selected by simple random sampling.

Diagnosis of T1DM was made according to the criteria of the International Society of Pediatric and Adolescent Diabetes (ISPAD) 2022 [13].

Exclusion criteria included children and adolescents with proven hyperlipidemia, hypertension, obesity, other autoimmune diseases, acute coronary syndrome, stroke, history of deep venous thrombosis or pulmonary embolism, and history of malignancy or coagulation disorders. Patients with hypocalcemia and those receiving treatment with aspirin, clopidogrel, warfarin, nonsteroidal anti-inflammatory drugs, or any drug that affects the coagulation system were also excluded.

#### Sample size calculation

Using the PASS 11.0 program & based on a study carried out by Sobczak et al. [2] a sample size of 92 participants in total, 46 patients & 46 controls is sufficient to detect a difference of 29.5% between groups in individual fibrin clot parameters given that power is 80% & confidence level 95% taking in consideration the 10% drop out rate.

The patient group was further subdivided into 2 groups according to the presence or absence of microangiopathy (17 patients with complications and 29 patients without complications).

### Ethical considerations

The study protocol was approved by the Ethical Committee of Ain Shams University with an approval number MS 185/2022 and written informed consent was obtained from all cases and their legal guardians before participation.

### Procedures

#### Clinical assessment

All enrolled participants were subjected to detailed medical history collection with special emphasis on demographic data, age at onset of diabetes, diabetes duration, and insulin therapy. Anthropometric measures assessment was done including weight in kilograms (Kg), height in centimeters (cm), and body mass index (BMI) calculation in kg/m2 plotting them on age and gender standard percentiles [14].

The modified magnesium deficiency questionnaire (MDQ)-10 was used by a single physician to assess the symptoms of magnesium deficiency. MDQ-10 is a validated 10 questions questionnaire that showed a significant correlation with hypomagnesemia; its total score range is 0–31, with a cut-off value of >5 [15].

Fundus examination was done for children and adolescents with T1DM by direct ophthalmoscope through dilated pupils for assessment of diabetic retinopathy [16]. A complete neurological examination was performed for children and adolescents with T1DM by a single-blinded neurologist. The Toronto Clinical Scoring System (TCSS) was adopted as a screening tool for diabetic peripheral neuropathy. It consists of three parts: symptom scores, reflex scores, and sensory test scores. The maximum score is 19 points [17].

#### Biochemical assessment

##### Blood sampling

About 5 ml of blood volume was withdrawn from each participant for the following investigations

a) Magnesium level:

Serum magnesium was assessed by the direct method in which a colored complex is measured at 520/800 nm at Beckman Coulter Au 480.

b) Glycosylated hemoglobin (HbA1c):

HbA1c measurement was carried out using the Tina-QuantR HbA1c kit supplied by Roche Diagnostics on Roche/Hitachi CobasR* c501 System (Roche Diagnostics International Ltd. CH-6343 Rotkreuz, Switzerland) based on turbidimetric inhibition immunoassay (TINIA).

c) PT / aPTT clotting:

A blood sample was placed in a clean tube containing sodium citrate. The citrated blood tube was centrifuged at 2500 rpm for 20 minutes and plasma was separated into a clean Eppendorf tube and used to immediately measure prothrombin time (PT= 11–13.5 seconds), partial thromboplastin time (PTT= 23–35 seconds) and calculate the INR (INR= patient PT/normal reference range of PT= 0.8–1.1). The coagulation profile included PT, PTT, and INR was performed by viscosity-based electromechanical detection systems using Stago STA4 Compact (Diagnostica Stago, France).

d) Serum levels of PAI-1:

Determination of serum levels of PAI-1 using ELISA based on double antibody sandwich tech (Bioassay Technology Laboratory; E1159HU, Shanghai Korain Biotech Co., Shanghai, China).

e) Fasting serum triglycerides (TG) and total cholesterol (TC) were assessed by quantitative enzymatic colorimetric technique (Bio Merieux-Diagnostic Chemicals Ltd., Charlottetown, CA, USA). Serum high-density lipoprotein (HDL) was measured by the phosphotungstate precipitation method (Bio Merieux kit, Marcyl’Etoile, Craponne, France). LDL cholesterol was calculated by Friedewald’s formula [18].

##### Urine sampling

Urinary albumin excretion in 3 consecutive early morning fasting spot urine samples was assayed for children and adolescents with T1DM by an immuno-turbidimetric method using the Beckman Colter AU 480 system (Beckman coulter, Inc. 250 s. Kraemer Blvd. Brea, CA92821, USA. Albuminuria was defined by an albumin excretion rate of > 30 mg/g creatinine, microalbuminuria as urinary albumin excretion 30–299 mg/g creatinine and macroalbuminuria as urinary albumin excretion ≥ 300 mg/g creatinine. Nephropathy was defined as having micro or macro-albuminuria [19].

#### Statistical analyses

Data were collected, revised, coded, and entered into the Statistical Package for Social Science (IBM SPSS) version 27. The quantitative data were presented as mean, standard deviations, and ranges when their distribution was found parametric. Qualitative variables were presented as numbers and percentages.

The comparison between the two groups regarding qualitative data was done by using the Chi-square test. The comparison between two independent groups with quantitative data and parametric distribution was done by using the independent t-test. Spearman correlation coefficients were used to assess the correlation between two quantitative parameters in the same group. ***Stepwise multiple linear regression analysis*** was performed to detect the most significant independent variables associated with hypomagnesemia among the studied children and adolescents with T1DM. The confidence interval was set to 95% and the margin of error accepted was set to 5%. So, the *p*value was considered significant at the level of <0.05.

## Results

This controlled cross-sectional study included 46 randomly selected children and adolescents with T1DM & 46 age and gender-matched healthy controls. There were no dropouts. The mean age of the studied children and adolescents with T1DM was 13.7 ±3.1 years, with a mean diabetes duration of 7.9±1.8 years. Their mean HbA1c % was 9.2 ±1.5%. They were all on basal bolus insulin regimen with a mean total insulin daily dose of 0.99 U/kg/day and self-monitoring of blood glucose 7 times daily. As regards microangiopathy, 17 had nephropathy (39.7%), one had nonproliferative diabetic retinopathy (2.2%) and two had both neuropathy and nephropathy (4.3%). None of the patients was on additional medications for the complications. The clinico-laboratory characteristics of the studied children and adolescents with T1DM are described in Table 1.

**Table 1:** Clinico-laboratory characteristics of the studied children and adolescents with T1DM.

	Children and adolescents with T1DM (*n*=46)	
Clinical:		
Gender	Male	21 (45.7%)
	Female	25 (54.3%)
Age (years)	Mean ± SD	13.7±3.1
	Range	8–18
Diabetes duration (years)	Mean ± SD	7.9±1.8
	Range	5–14
Daily insulin dose (unit/kg/day)	Mean ± SD	0.99±0.67
	Range	0.33–2.4
Laboratory:		
HbA1c %	Mean ± SD	9.2±1.5
	Range	6.9–14
Cholesterol (mg/dl)	Mean ± SD	173.0±41.6
	Range	122.0–270.0
Triglycerides (mg/dl)	Mean ± SD	92.1±33.8
	Range	40.0–166.0
HDL (mg/dl)	Mean ± SD	55.3±14.9
	Range	31.0–87.0
LDL-c (mg/dl)	Mean ± SD	100.9±23.1
	Range	60.0–160.0
Magnesium (mg/dL)	Mean ± SD	1.3±0.1
	Range	0.6-1.5
PT (seconds)	Mean ± SD	11.1±0.2
	Range	7-11.5
APTT (seconds)	Mean ± SD	21.2±1.9
	Range	15-24
PAI-1 (ng/ml)	Mean ± SD	10.3±4.9
	Range	3.7–24

*T1DM* Type 1 diabetes mellitus, *HbA1c* Glycated hemoglobin, *HDL* high-density lipoproteins, *LDL-c* low-density lipoproteins, *PT* prothrombin time, *aPTT* activated partial thromboplastin time, *PAI-1* plasminogen activator inhibitor-1.

### Serum magnesium among children and adolescents with T1DM

The studied children and adolescents with T1DM exhibited clinical manifestations of hypomagnesemia using the MDQ-10 in 28.3%, including tingling in 21.7% and muscle cramps in 26.1%. The mean serum magnesium of the studied children and adolescents with T1DM was 1.3 mg/dl, range 0.6–1.5. Clinical manifestations of hypomagnesemia were significantly more common among children and adolescents with T1DM than controls (p<0.001), which goes in concordance with the serum magnesium that was significantly lower among children and adolescents with T1DM than controls (*p*<0.001), Table 2. Moreover, serum magnesium was found to be negatively correlated with insulin daily dose (*p*=0.042) among the studied children and adolescents with T1DM, Table 3.

**Table 2:** Comparison between children and adolescents with T1DM and controls regarding various clinico-laboratory parameters.

		Children and adolescents with T1DM	Control		
		(*n*=46)	(*n*=46)	Test	*p* value
Gender	Male	21 (45.7%)	18 (39.1%)	X^2^=0.41	0.520
	Female	25 (54.3%)	28 (60.9%)		
Age (years)	Mean ± SD	13.7±3.1	12.9±2.9	t=0.98	0.260
	Range	8–18	7–18		
Clinical:					
MDQ-10	Deficiency	13 (28.3%)	0 (0.0%)	X^2^=15.1	<0.001*
Tingling	Positive	10 (21.7%)	0 (0.0%)	X^2^=11.2	<0.001*
Muscle cramps	Positive	12 (26.1%)	0 (0.0%)	X^2^=13.8	<0.001*
Laboratory:					
Magnesium (mg/dL)	Mean ± SD	1.3 ±0.1	2.0 ±0.2 1.7-2.4	t=20.8	<0.001*
	Range	0.6-1.5			
PT (seconds)	Mean ± SD	11.1 ±0.2	12.3 ±0.3 12-13	t=21.6	<0.001*
	Range	7–11.5			
APTT (seconds)	Mean ± SD	21.2 ±1.9	31.1 ±2.7 25-35	t=17.6	<0.001*
	Range	15–24			
PAI-I (ng/ml)	Mean ± SD	10.3 ±4.9	2.8 ±1.3 1.5-10.5	t=9.95	<0.001*
	Range	3.7–24			
Cholesterol (mg/dl)	Mean ± SD	173.0 ±41.6	101.9 ±10.7 85.0-123.0	t=11.23	<0.001*
	Range	122.0–270.0			
Triglycerides (mg/dl)	Mean ± SD	92.1 ±33.8	66.4 ±18.3 40.0-91.0	t=4.53	<0.001*
	Range	40.0–166.0			
HDL (mg/dl)	Mean ± SD	55.3 ±14.9	67.4 ±8.11	t=-4.81	<0.001*
	Range	31.0–87.0	54.0-84.0		
LDL-c (mg/dl)	Mean ± SD	100.9 ±23.1	81.5 ±14.1	t=4.84	<0.001*
	Range	60.0-160.0	65.0-110.0		

X^2^: Chi square test, t: Student t-test, Bold and asterisk (*): significant.

*T1DM* Type 1 diabetes mellitus, *MDQ-10* magnesium deficiency questionnaire-10, *HDL* high-density lipoproteins, *LDL-c* low density lipoproteins, *PT*prothrombin time, *aPTT* activated partial thromboplastin time, *PAI-1* plasminogen activator inhibitor-1.

**Table 3:** Correlations between serum magnesium and various clinico-laboratory data among the studied children and adolescents with T1DM and controls.

	Serum magnesium			
	Children and adolescents with T1DM and controls (*n*=46)		Controls (*n*=46)	
	r	*P* value	r	*P* value
Age (years)	0.223	0.137	0.004	0.976
Diabetes Duration (years)	−0.247	0.098	-	-
Insulin daily dose (unit/kg/day)	−0.244	0.042*	-	-
PT (seconds)	0.856	<0.001*	0.181	0.288
APTT (seconds)	0.808	<0.001*	0.070	0.964
PAI-I (ng/ml)	−0.643	<0.001*	−0.120	0.325
HbA1c %	−0.294	0.023*	-	-
Urinary micro albumin mg/gm creatinine	−0.286	0.036*	-	-
Cholesterol (mg/dl)	−0.045	0.769	0.001	0.995
Triglycerides (mg/dl)	−0.467	0.001*	0.141	0.351
HDL (mg/dl)	0.382	0.009*	-0.004	0.982
LDL (mg/dl)	−0.100	0.510	0.048*	0.751

Spearman correlation co-efficient, r: correlation co-efficient, Bold and asterisk (*): significant.

*PT* Prothrombin time, *APTT* Activated Partial Thromboplastin Time, *PAI-I* Plasminogen Activator Inhibitor Type-1, *HDL* High-density lipoprotein, *LDL* Low-density lipoprotein.

### Coagulopathy among children and adolescents with T1DM

As shown in Table 2, children and adolescents with T1DM had significantly lower PT and aPTT levels with significantly higher PAI-1 levels than controls (*p*<0.001).

### Hypomagnesemia and metabolic control (microangiopathy and glycemic control)

**Fig. 1:**
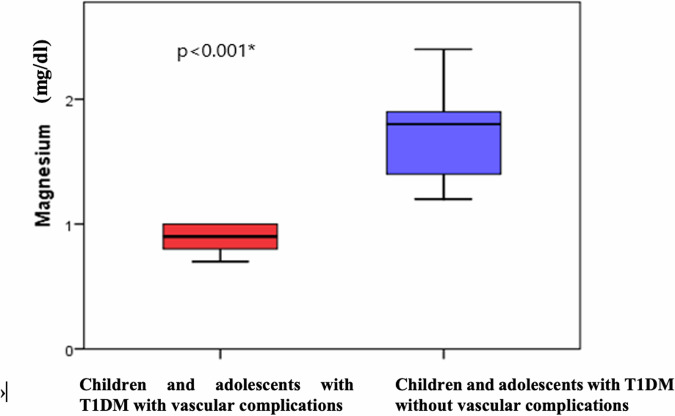
Comparison between children and adolescents with T1DM with and without diabetic vascular complications as regarding serum magnesium level.

Regarding magnesium’s relation to microangiopathy, children and adolescents with T1DM having microangiopathy were found to have significantly higher numbness (*p*=0.019), tingling (*p*=0.012), and muscle cramps (*p*<0.001) with significantly lower serum magnesium than those without microangiopathy (*p*<0.001), Fig. 1 and Table 4. Moreover, magnesium was found to be negatively correlated to urinary microalbumin (*p*=0.036), HbA1c (*p*=0.023) triglycerides (*p*=0.001) and positively correlated to HDL-cholesterol (*p*=0.009) among children and adolescents with T1DM, Table 3.

**Table 4:** Comparison between children and adolescents with T1DM with and without vascular complications regarding various clinico-laboratory parameters.

		Children and adolescents with T1DM			
		With vascular complications (*n*=17)	Without vascular complications (*n*=29)	Test	*P* value
Symptoms of magnesium deficiency					
MDQ-1	Deficiency	7 38.9%	6 21.4%	X^2^=2.6	0.019*
Tingling	Positive	6 33.3%	4 14.3%	X^2^=2.2	0.012*
Muscle cramps	Positive	8 44.4%	4 14.3%	X^2^=5.1	<0.001*
Laboratory parameters					
Magnesium (mg/dL)	Mean ± SD	0.9±0.1	1.3±0.1	t=14.1	<0.001*
	Range	0.7-1.0	1.2-1.5		
PT (seconds)	Mean ± SD	8.2±2.0	11.0±0.3	t=7.3	<0.001*
	Range	7-10	10-11.5		
aPTT (seconds)	Mean ± SD	15.7±0.8	21.3±1.8	t=12.3	<0.001*
	Range	15-18	18-24		
PAI-I (ng/ml)	Mean ± SD	11.0±5.2	9.3±4.3	t=4.6	<0.001*
	Range	4-21	3.7-24		

X^2^: Chi square test, t: Student t-test, *MDQ-10* magnesium deficiency questionnaire-10, *PT* Prothrombin time, *aPTT* Activated Partial Thromboplastin Time, *PAI-I* Plasminogen Activator Inhibitor Type-1.

Bold and asterisk (*): significant.

### Coagulopathy and metabolic control (microangiopathy and glycemic control)

**Fig. 2:**
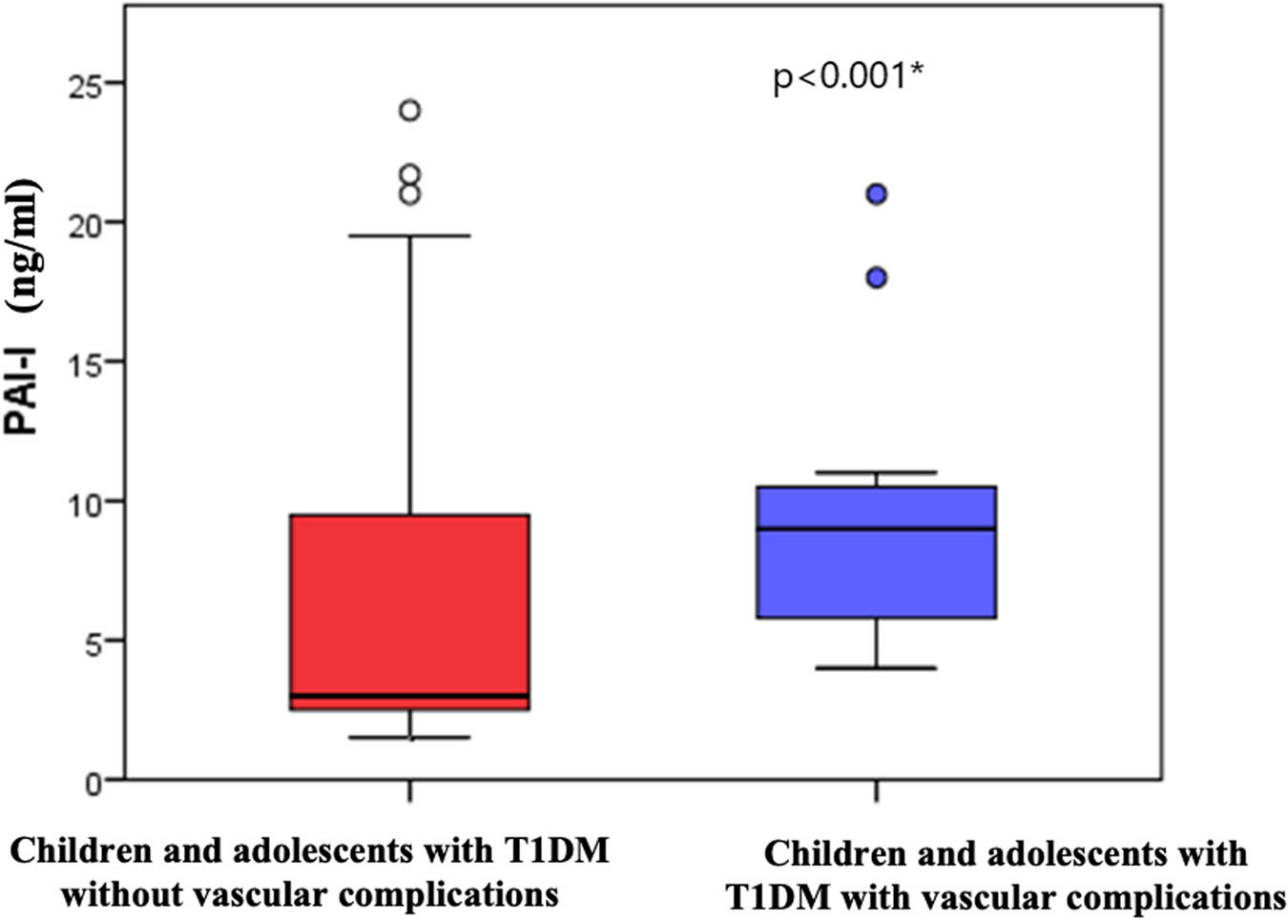
Comparison between children and adolescents with T1DM with and without diabetic vascular complications as regarding plasminogen activator inhibitor-1 (PAI-1) level.

As for coagulopathy, children and adolescents with T1DM having microangiopathy were found to have significantly lower PT and aPTT with significantly higher PAI-1 than those without microangiopathy (*p*<0.001), Fig. 2 and Table 4.

### Hypomagnesemia and coagulopathy

Interestingly, serum magnesium was found to be positively correlated with PT and aPTT and negatively correlated with PAI-1 among the studied children and adolescents with T1DM (*p<*0.001) but not in controls (*p*>0.05), Table 3.

Moreover, multivariate logistic regression analysis revealed that serum magnesium is independently associated with diabetes duration (*p*=0.015), HbA1c (*p*=0.001), PT (*P*=0.034), aPTT (*p*=0.001), urinary microalbumin (*p*=0.005) and PAI-1 (*P*=0.001) among the studied children and adolescents with T1DM, Table 5.

**Table 5:** Linear Regression analysis for factors associated with hypomagnesemia among the studied children and adolescents with T1DM.

	Unstandardized Coefficients		Standardized Coefficients	t	Sig.	95.0% Confidence Interval for B	
	B	Std. Error	Beta			Lower Bound	Upper Bound
(Constant)	0.196	0.254		0.771	0.446	−0.321	0.713
Diabetes duration (years)	−0.052	0.011	−0.293	−2.915	0.015*	−0.075	0.001
HBA1C %	−0.206	0.115	−0.434	−3.381	0.001*	−0.337	−0.026
PT (sec)	0.033	0.016	0.297	2.202	0.034*	−0.001	0.066
aPTT (sec)	0.042	0.012	0.526	3.633	0.001*	0.019	0.066
Cholesterol (mg/dl)	0.001	0.001	0.133	1.417	0.166	0.000	0.002
Triglycerides (mg/dl)	−0.001	0.001	−0.098	−0.911	0.369	−0.002	0.001
HDL (mg/dl)	0.002	0.002	0.096	1.014	0.318	−0.002	0.005
LDL (mg/dl)	−0.001	0.001	−0.108	−1.052	0.301	−0.003	0.001
Urinary microalbumin	−0.211	0.103	−0.343	−2.866	0.005*	−0.460	−0.112
PAI-1	0.276	0.093	0.511	4.132	0.001*	0.011	0.543

Bold and asterisk (*): significant.

*PT* Prothrombin time, *APTT* Activated Partial Thromboplastin Time, *PAI-I*Plasminogen Activator Inhibitor Type-1, *HDL* High-density lipoprotein, *LDL* Low-density lipoprotein.

## Discussion

Diabetic microangiopathy represent significant morbidity among people with T1DM. Identifying the patho-mechanism of such complications and its determining factors is of utmost importance. Glycemic variability and chronic hyperglycemia among people with diabetes were found to be accompanied by an increased risk of atherosclerosis, vascular complications, and hypercoagulability [20]. This is thought to result from endothelial dysfunction, enhanced platelets activity, abnormal coagulation factors activity, together with impairment in the fibrinolytic system [21, 22].

Regarding the coagulation system, shortened PT and aPTT are known to reflect a hypercoagulable state, which is potentially associated with increased thrombotic risk and vascular complications [23, 24]. In the current study, children and adolescents with T1DM were found to have significantly reduced PT and aPTT levels than controls. This reduction in PT and aPTT was more evident in those having diabetic microangiopathy than those without. In line with these results, Zhao et al. and Lippi et al. found that aPTT values in people with T2DM, high risk of diabetes, or impaired fasting glucose were significantly shorter than controls suggesting that aPTT might be a risk predictor of thrombosis and microangiopathy among people with diabetes [25]. Similarly, Sapkota et al. and Kim and colleagues reported shortening of PT and aPTT in adults with T1DM in comparison to healthy controls [26, 27]. On the contrary, Binay et al. found no difference in PT, and aPTT between children with T1DM and healthy controls [28]; which could be attributed to the shorter diabetes duration in their study (3.15 ± 2.49 years) versus (7.9 ± 1.8 years) in the current study and the absence of microangiopathy in their cohort.

As for the fibrinolytic system, diabetes was found to be associated with hypo-fibrinolysis even early in the course of the disease contributing directly to the enhanced microvascular risk in this population [29]. A central regulator of fibrinolysis that is affected in diabetes is the plasminogen activator inhibitor PAI-1 [30]. In the current study, children and adolescents with T1DM were found to have significantly higher PAI-1 than controls. This elevated PAI-1 level was even higher in those having microangiopathy than those without. In line with these results, Adly and colleagues found elevated PAI-1 levels in children with T1DM especially those having microangiopathy [30]. Similarly, Aleman and colleagues found significantly higher PAI-1 levels among children with T1DM without microangiopathy, that were correlated with HbA1c and fibrinogen levels [31]. In contrast, Agren and colleagues found that despite the increased incorporation of antiplasmin into the fibrin network among adults with T1DM, they had lower PAI-1 levels than controls. However, among their cohort with T1DM; those with T1DM on statins (probably due to diabetic microangiopathy) had higher levels of PAI-1 than those not on statins [1]. The increased PAI-1 in T1DM is supported by in vitro studies that found that increased glucose concentration enhances PAI-1 expression in both endothelial and vascular smooth muscle cells [32].

Magnesium plays a crucial role in normal cellular physiology and metabolism, being a cofactor of multiple enzymes, regulator of energy generation and ion channels. Moreover, it has a role in vascular tone regulation, atherogenesis, and endothelial proliferation. Magnesium deficiency was found to increase endothelial susceptibility to oxidative stress promoting endothelial dysfunction, reduce fibrinolysis, and increase coagulation [33].

Hypomagnesemia is a common morbidity in people with T2DM. It is reported to be 10-fold more common in people with T2DM than the general population. In T2DM, hypomagnesemia has been linked to insulin resistance, poor glycemic control and diabetic microangiopathy [34]. In T1DM, hypomagnesemia is thought to be less common and its relation to diabetic microangiopathy has not been thoroughly investigated. The mean serum magnesium of the studied children and adolescents with T1DM was 1.3 mg/dl, range 0.6-1.5, with only 2 children having hypomagnesemia (4.3%). This goes in concordance with recent studies on large cohorts with T1DM that found comparable prevalence of hypomagnesemia among people with T1DM 0 f 2.9% and 4.3% respectively [35, 36]. Although frank hypomagnesemia was not prevalent among the studied children and adolescents with T1DM; their mean magnesium level was significantly lower than controls. This goes in line with Fritzen and colleagues who reported significantly lower mean plasma magnesium concentration in people with T1DM compared with age-matched controls [36].

Moreover, children and adolescents with T1DM having microangiopathy were found to have significantly lower serum magnesium than those without microangiopathy. In addition, magnesium was found to be negatively correlated with urinary microalbumin, HbA1c, triglycerides and positively correlated with HDL cholesterol among children and adolescents with T1DM indicating that even minimal reduction in magnesium concentration might have negative consequences. These findings are in line with data by Van Dijk and colleagues who found that markers of oxidative stress exhibit a negative correlation with magnesium level [5] and with older studies showing a significant association between low magnesium concentrations and poor glycemic control and the presence of diabetic microangiopathy [37–39]. Postulated patho-mechanisms linking low magnesium concentrations with diabetic vascular complications include higher levels of tumor necrosis factor-alpha and increased formation of advanced glycosylation end products [9, 40]. In the current study, we suggest an association between low magnesium concentration; hypofibrinolysis, reduced PT and PTT, poor glycemic control and diabetic microangiopathy; rendering magnesium a possible therapeutic target for prevention and treatment of diabetic microangiopathy.

In support with this postulation, serum magnesium was found to be positively correlated with PT, aPTT and HDL, and negatively correlated with PAI-1, HbA1c, triglycerides among the children and adolescents with T1DM, but not in controls which suggest its role in diabetic microangiopathy.

## Study limitations

One limitation of this study is its cross-sectional nature which could not imply causality and the small sample size. Another limitation is the poor control of most of the studied children and adolescents with T1DM, given the use of simple random sampling (only 4 were well controlled), that does not give us an idea about the magnesium level, coagulation and fibrinolytic status of well-controlled children and adolescents with T1DM. Therefore, larger longitudinal studies, including well-controlled children and adolescents with T1DM are needed to identify the mechanistic link between T1DM, hypomagnesemia, coagulation disorders, hypo-fibrinolysis, and diabetic microangiopathy.

## Conclusion

In conclusion, children and adolescents with T1DM have lower magnesium, PT, and aPTT and higher PAI-1 levels than controls. These findings are more pronounced among those having microangiopathy than those without. Moreover, hypomagnesemia is independently associated with poor glycemic control, coagulopathy, hypofibrinolysis, and diabetic microangiopathy among children and adolescents with T1DM. Thus, hypomagnesemia might have a mechanistic role in the pathogenesis of coagulation disorders and hypo-fibrinolysis among children and adolescents with T1DM contributing to diabetic microangiopathy. Hence, providing therapeutic modalities targeting hypomagnesemia, PAI-1, PT, and aPTT abnormalities could provide insight into the prevention and treatment of diabetic microangiopathy. Magnesium supplementation combined with standard insulin therapy in pediatric patients with T1DM is recommended for better glycemic control and prevention of diabetic microangiopathy. Further larger longitudinal studies are mandatory to clarify the effect of magnesium supplementation on the control of T1DM, as well as its effect on diabetic microangiopathy.

## Data Availability

The data sets used and/or analyzed during the current study are available from the corresponding author upon reasonable request.
